# Supplementary material for: A role of the Nse4 kleisin and Nse1/Nse3 KITE subunits in the ATPase cycle of SMC5/6
Source: Sci Rep. 2020 Jun 16;10:9694. doi: 10.1038/s41598-020-66647-w (PMC7297730; doi:10.1038/s41598-020-66647-w)
Supplement: Supplementary file 1 — Supplementary Information. [file 41598_2020_66647_MOESM1_ESM.pdf]

A role of the Nse4 kleisin and Nse1/Nse3 KITE subunits in the ATPase cycle of SMC5/6

Vondrova L. [1], Kolesar P. [1], Adamus M. [2], Nociar M. [1], Oliver A.W. [3], Palecek J.J. [1,2*]

[1] National Centre for Biomolecular Research, Faculty of Science, Masaryk University, Kamenice 5, 62500 Brno, Czech Republic

[2] Mendel Centre for Plant Genomics and Proteomics, Central European Institute of Technology, Masaryk University, Kamenice 5, 62500 Brno, Czech Republic

[3] Genome Damage and Stability Centre, School of Life Sciences, University of Sussex, Falmer, Brighton BN1 9RQ, United Kingdom

SUPPLEMETARY INFORMATION

METHODS

PEPSCAN-ELISA

The PEPSACAN was performed as described previously[1] with a peptide library (Mimotopes, Australia) of the aa 875-1024 region of the *S. pombe* Smc6 protein (Supplementary Table 1). The library was linked to biotin via an additional peptide spacer of Ser–Gly–Ser–Gly. The peptides were prebound to ELISA plates (coated with streptavidin) and washed three times with binding buffer (PBS with 0.5% Nonidet NP40). Then, *S. pombe* His-S-Nse4 (aa1-150) protein was added and incubated overnight. Unbound protein was washed (three times) and the peptide-bound Nse4 protein was quantified using anti-His (Sigma H1029, 1:10000) and anti-mouse HRP-conjugated (Sigma A0168, 1:10000) antibodies, respectively. *H. sapiens* His-TRF2 (aa1-542) protein was used as a negative control in the same way[2].

Protein expression analysis

Yeast cells were grown to OD~1 and lysed by incubation in 0.1 M NaOH for 5 min and boiling in SDS Laemmli buffer (62.5 mM Tris–HCl, 2% SDS, 5% β-mercaptoethanol, 10% glycerol, 0.002% bromophenol blue; [3]). Samples were separated by 12% SDS-PAGE and immunoblotted with anti-Gal4-AD (Sigma Aldrich - G9293) or anti-Gal4-BD (Abcam - ab135397) antibodies.

The other methods are described in the main text.

FIGURE LEGENDS

Supplementary Figure S1. Nse4 binds neck region of SMC6.

Peptide library (**A**) and multi-component yeast two-hybrid systems (**B-D**) were employed to determine the SMC6 region and residues binding to Nse4. (**A**) Quantification of relative binding of the Nse4(1-150) protein (Nse4; red columns) to the SMC6 synthetic peptides (listed in Supplementary Table 1) using the PEPSCAN-ELISA method. The SMC6(aa960-984) peptide exhibits the highest affinity and specificity to Nse4. Results show mean $\pm$ SEM of 3 independent measurements. His-TRF2 protein (TRF2; white column) was used in the control experiment. (**B-D**) To identify the Nse4-binding residues, stability of the following SMC6 mutant complexes was tested: SMC6-Nse4-Nse3-Nse1 (**B**), SMC6-Nse4-SMC5 (**C**) and SMC6-Nse5-Nse6 (**D**). (**B**) The full-length hybrid SMC6 (fused to Gal4AD domain) and Nse4 (fused to Gal4BD domain) were co-transformed together with the Nse1-Nse3 construct (p416ADH1 vector) into the PJ69 cells. Formation and stability of the SMC6-Nse4-Nse3-Nse1 complex was scored by growth of the yeast PJ69 transformants on the plates without Leu, Trp, Ura and His, containing 0.3 mM 3-Amino-1,2,4-triazole (-L,T,U,H, 0.3AT panel). (**C**) Similarly, the full-length SMC6 (fused to Gal4AD domain) and SMC5 (fused to Gal4BD domain) were co-transformed together with the Nse4 full-length construct (in p416ADH1 vector) and stability of the SMC5-Nse4-SMC6 complex was scored on the plates containing 0.5 mM 3-Amino-1,2,4-triazole (-L,T,U,H, 0.5AT panel). The L964A, L965A, L968A, E969A, L972A and R975A mutations reduce stability of the SMC6-Nse4 complexes (**B** and **C**). (**D**) In the control experiment, the same mutations were introduced to the SMC6-Nse5-Nse6 complex (constituted of the full-length Gal4AD-SMC6, Gal4BD-Nse5 and non-hybrid Nse6). Stability of the SMC6-Nse5-Nse6 complex was scored on plates containing 3 mM 3-Amino-1,2,4-triazole (-L,T,U,H, 3AT panel). The L964A, L968A and E969A mutations affected all the SMC6 complexes (**B-D**). In contrast, the highly conserved L965, L972 and L975 SMC6 residues were required

specifically for the binding to Nse4 (**B** and **C**). Wild-type (WT) or mutant versions of SMC6 are labelled in blue below the panels; "-",the empty vector control; "+", co-transformed construct (as indicated at the left side). Growth of the transformants was verified on the control plates without leucine, tryptophan and uracil (-L,T,U). All Y2H tests were repeated at least 3 times. (**E**) The expression of the WT and mutant Gal4AD-SMC6 hybrid proteins was compared on the western blots. Control lane (cont): the empty pGADT7 vector.

Supplementary Figure S2. Binding of the Nse4 helix H3 to SMC6.

(**A**) The full-length hybrid SMC6 (fused to Gal4AD domain) and Nse4 (fused to Gal4BD domain) were co-transformed together with the Nse1-Nse3 construct (p416ADH1 vector) into the PJ69 cells. Formation and stability of the SMC6-Nse4-Nse3-Nse1 complex was scored by 4Y2H on the plates containing 0.5 mM 3-Amino-1,2,4-triazole (-L,T,U,H, 0.5 AT panel; further details as in Fig. S1). (**B**) The expression levels of the WT and mutant Gal4BD-myc-Nse4 hybrid proteins (MW cca 60 kDa) are comparable; control lane (cont): the pGBKT7 vector expressing the Gal4BD-myc protein (MW cca 20 kDa). (**C**) The SMC3-Scc1 crystal structure 4UX3 (left) representing the νSMC-kleisin binding mode and the SMC6-Nse4 model (right) based on our protein-protein interaction analysis.

Supplementary Figure S3. The SMC5-SMC6-Nse4-Nse3-Nse1 instability is dependent on the ATP binding and SMC5-SMC6 head dimerization.

(**A-B**) Alignments of the conserved SMC head motifs mutated in the SMC5 and SMC6 constructs. Arrows point to the positions of the fission yeast SMC5/K57I mutation in the Walker A motif disturbing ATP binding (**A**), the SMC6/S1045R mutation in the Signature motif abrogating ATP-mediated SMC5-SMC6 head dimerization (**B**), and the SMC5/E995Q mutation in the Walker B motif inhibiting ATP hydrolysis (**B**), respectively. The SMC

homologs are from *Bacilus subtilis* (*B.s.*), *Schizosaccharomyces pombe* (*S.p.*), *Homo sapiens* (*H.s.*). Amino acid shading represents the following conserved amino acids: *dark green*, hydrophobic and aromatic; *light green*, polar; *pink*, basic; *blue*, acidic. (**C**) Stability of the SMC5-SMC6-Nse4 (columns 1-6) and SMC5-SMC6-Nse4-Nse3-Nse1 (columns 7-12) complexes was scored on the plates containing increasing concentrations of 3-Amino-1,2,4-triazole (AT). The SMC5/K57I (KI) mutation disturbing ATP binding and SMC6/S1045R (SR) mutation abrogating ATP-mediated SMC5-SMC6 head dimerization has no effect on the SMC5-SMC6-Nse4 complex irrespective of the SMC5/E995Q mutation (columns 3-6). In contrast, destabilizing effect of the SMC5/E995Q mutation was fully supressed by both KI and SR mutations in the SMC5-SMC6-Nse4-Nse3-Nse1 complex (compare columns 8, 10 and 12), suggesting that the instability is caused by the ATP binding and ATP-mediated head dimerization. Mutant versions of SMC5 and SMC6 are labelled in grey and blue, respectively (further details as in Suppl. Fig. S1). (**D**) The expression of the WT and mutant Gal4BD-SMC5 hybrid proteins (MW cca 160 kDa) was compared on the western blot; control lane (nr. 1): the pGBKT7 vector expressing the Gal4BD protein (MW cca 20 kDa).

Supplementary Figure S4. Extension of the Nse4 linker size affects stability of both ATP-free and ATP-bound core complexes.

(**A**) Alignment of the end of the Nse4 linker and C-terminal WHD domain. The Nse4 orthologs are from *Schizosaccharomyces pombe* (*S.p.*), *Aspergillus nidulans* (*A.n.*), *Aspergillus clavatus* (*A.c.*), *S. cerevisiae* (*S.c.*), *Danio rerio* (*D.r.*), *Xenopus laevis* (*X.l.*), *Ornithorhynchus anatinus* (*O.a.*), *Loxodonta africana* (*L.a.*), *Monodelphis domestica* (*M.d.*), *Dasypus novemcinctus* (*D.n.*), *Mus musculus* (*M.m.*), *Homo sapiens* (*H.s.*). The 30aa extension was inserted at the end of the Nse4 linker (after aa174) and in front of the conserved winged-helix domain (WHD; further details as in the Suppl. Fig. S3). (**B**) In the control experiments, the 30aa, 60aa and 90aa

extensions do not affect the stability of either Nse3-Nse4 interaction (columns 1-4) or Nse1-Nse3-Nse4 subcomplex (columns 5-8). (**C**) In the ATP-free core complexes, the 60aa and 90aa extensions gradually compromise the complex stability (columns 5 and 7), while the stability of the 30aa-extension containing complex is similar to the WT complex (columns 1 and 3). In the ATP-bound complexes, the 30aa and 60aa extensions partially suppress the ATP-imposed constraint (compare columns 2, 4 and 6). With the 90aa long extension, the stability of both ATP-free and ATP-bound complexes is similar, suggesting that high flexibility of the linker affects both its proper shape and its mechanical constraint. (**D**) Schematic summary of the panel **C** results.

REFERENCES

1 Guerineau, M. *et al.* Analysis of the Nse3/MAGE-Binding Domain of the Nse4/EID Family Proteins. *PLoS One* **7**, e35813. (2012).

2 Nečasová, I., Janoušková, E., Klumpler, T. & Hofr, C. Basic domain of telomere guardian TRF2 reduces D-loop unwinding whereas Rap1 restores it. *Nucleic Acids Research* **45**, 12170-12180, doi:10.1093/nar/gkx812 (2017).

3 Kushnirov, V. V. Rapid and reliable protein extraction from yeast. *Yeast* **16**, 857-860, doi:10.1002/1097-0061(20000630)16:9<857::aid-yea561>3.0.co;2-b (2000).

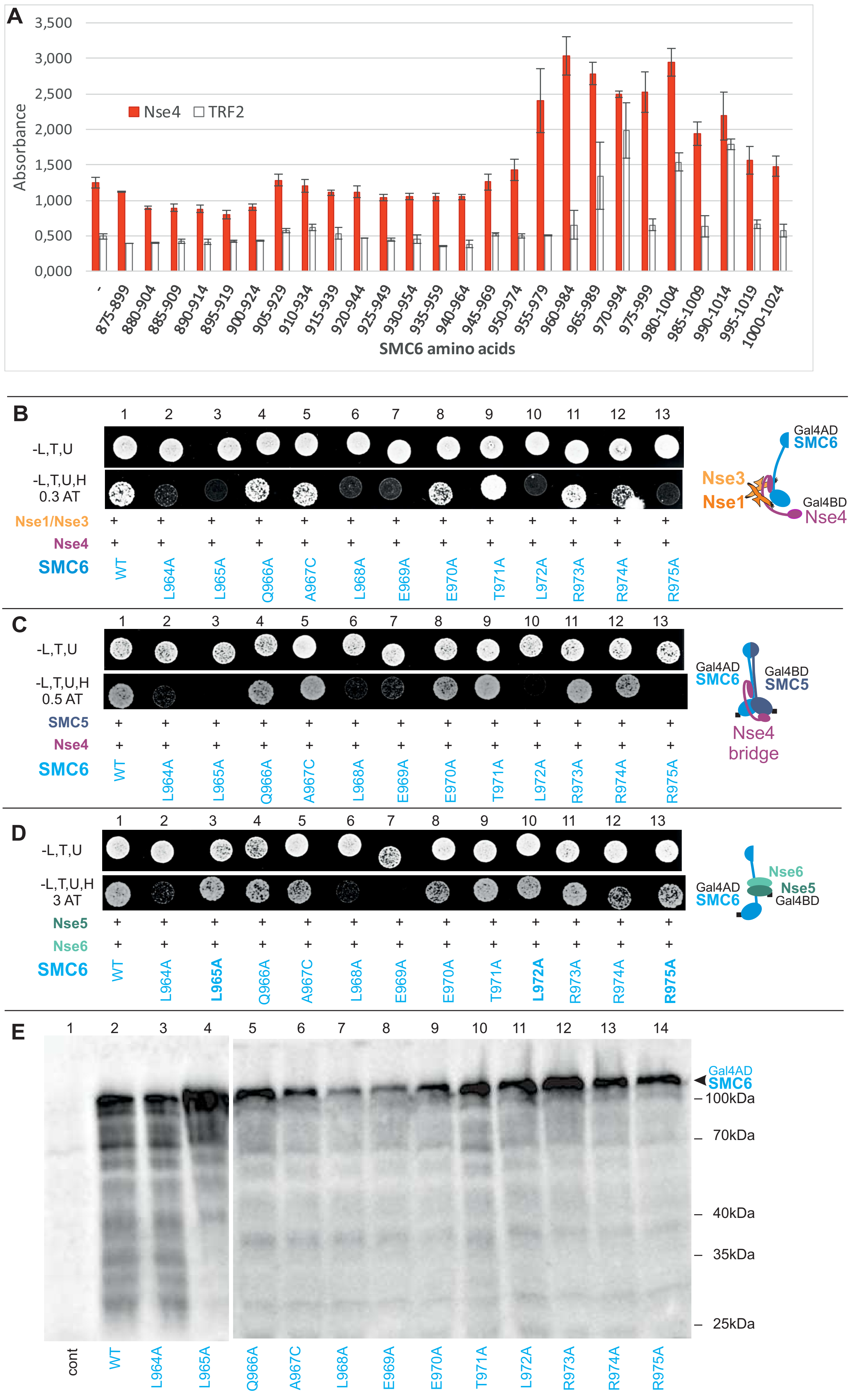
A
Absorbance
3,500
3,000
2,500
2,000
1,500
1,000
0,500
0,000
Nse4 TRF2
-
875-899
880-904
885-909
890-914
895-919
900-924
905-929
910-934
915-939
920-944
925-949
930-954
935-959
940-964
945-969
950-974
955-979
960-984
965-989
970-994
975-999
980-1004
985-1009
990-1014
995-1019
1000-1024
SMC6 amino acids
B
-L,T,U
-L,T,U,H
0.3 AT
Nse1/Nse3
Nse4
SMC6
WT
L964A
L965A
Q966A
A967C
L968A
E969A
E970A
T971A
L972A
R973A
R974A
R975A
Gal4AD
SMC6
Nse3
Nse1
Gal4BD
Nse4
C
-L,T,U
-L,T,U,H
0.5 AT
SMC5
Nse4
SMC6
Gal4AD
SMC6
Gal4BD
SMC5
Nse4
bridge
D
-L,T,U
-L,T,U,H
3 AT
Nse5
Nse6
SMC6
Nse6
Nse5
Gal4AD
SMC6
Gal4BD
E
Gal4AD
SMC6
100kDa
70kDa
40kDa
35kDa
25kDa
cont

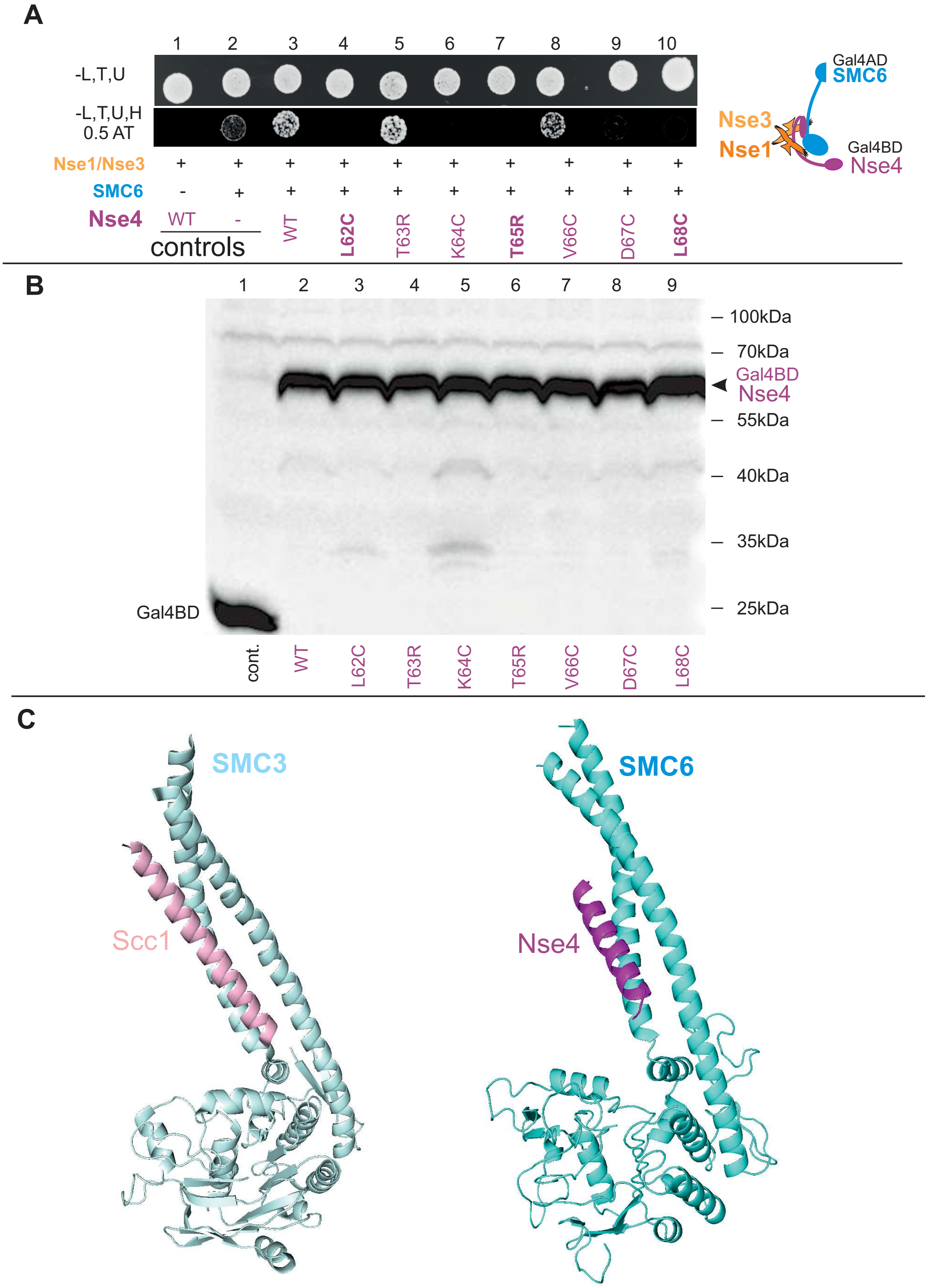
A
1 2 3 4 5 6 7 8 9 10
-L,T,U
-L,T,U,H 0.5 AT
Nse1/Nse3 + + + + + + + + + +
SMC6 - + + + + + + + + +
Nse4 WT - WT L62C T63R K64C T65R V66C D67C L68C
controls
Gal4AD
SMC6
Nse3
Nse1
Gal4BD
Nse4
B
1 2 3 4 5 6 7 8 9
100kDa
70kDa
Gal4BD
Nse4
55kDa
40kDa
35kDa
25kDa
Gal4BD
cont. WT L62C T63R K64C T65R V66C D67C L68C
C
SMC3
Scc1
SMC6
Nse4

S3

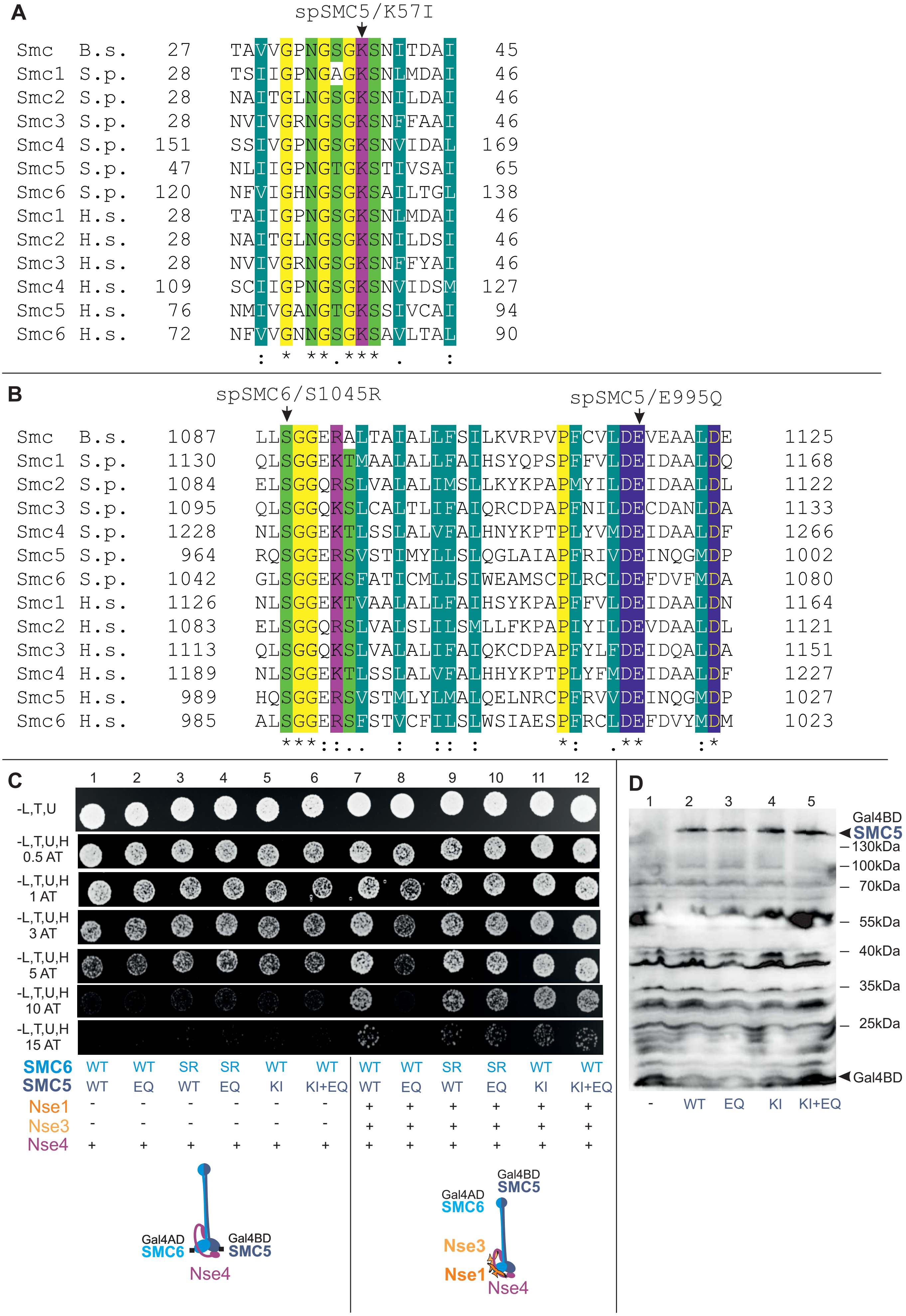
A
spSMC5/K57I
Smc B.s. 27 TAVVGPNGSGKSNITDAI 45
Smc1 S.p. 28 TSIIGPNGAGKSNLMDAI 46
Smc2 S.p. 28 NAITGLNGSGKSNILDAI 46
Smc3 S.p. 28 NVIVGRNGSGKSNFFAAI 46
Smc4 S.p. 151 SSIVGPNGSGKSNVIDAL 169
Smc5 S.p. 47 NLIIGPNGTGKSTIVSAI 65
Smc6 S.p. 120 NFVIGHNGSGKSAILTGL 138
Smc1 H.s. 28 TAIIGPNGSGKSNLMDAI 46
Smc2 H.s. 28 NAITGLNGSGKSNILDSI 46
Smc3 H.s. 28 NVIVGRNGSGKSNFFYAI 46
Smc4 H.s. 109 SCIIGPNGSGKSNVIDSM 127
Smc5 H.s. 76 NMIVGANGTGKSSIVCAI 94
Smc6 H.s. 72 NFVVGNNGSGKSAVLTAL 90
: * **.*** . :
B
spSMC6/S1045R
spSMC5/E995Q
Smc B.s. 1087 LLSGGERALTAIALLFSILKVRPVPFCVLDEVEAALDE 1125
Smc1 S.p. 1130 QLSGGEKTMAALALLFAIHSYQPSPFFVLDEIDAALDQ 1168
Smc2 S.p. 1084 ELSGGQRSLVALALIMSLLKYKPAPMYILDEIDAALDL 1122
Smc3 S.p. 1095 QLSGGQKSLCALTLIFAIQRCDPAPFNILDECDANLDA 1133
Smc4 S.p. 1228 NLSGGEKTLSSLALVFALHNYKPTPLYVMDEIDAALDF 1266
Smc5 S.p. 964 RQSGGERSVSTIMYLLSLQGLAIAPFRIVDEINQGMDP 1002
Smc6 S.p. 1042 GLSGGEKSFATICMLLSIWEAMSCPLRCLDEFDVFMDA 1080
Smc1 H.s. 1126 NLSGGEKTVAALALLFAIHSYKPAPFFVLDEIDAALDN 1164
Smc2 H.s. 1083 ELSGGQRSLVALSLILSMLLFKPAPIYILDEVDAALDL 1121
Smc3 H.s. 1113 QLSGGQKSLVALALIFAIQKCDPAPFYLFDEIDQALDA 1151
Smc4 H.s. 1189 NLSGGEKTLSSLALVFALHHYKPTPLYFMDEIDAALDF 1227
Smc5 H.s. 989 HQSGGERSVSTMLYLMALQELNRCPFRVVDEINQGMDP 1027
Smc6 H.s. 985 ALSGGERSFSTVCFILSLWSIAESPFRCLDEFDVYMDM 1023
***::.. : :: : *: .** :*
C
1 2 3 4 5 6 7 8 9 10 11 12
-L,T,U
-L,T,U,H 0.5 AT
-L,T,U,H 1 AT
-L,T,U,H 3 AT
-L,T,U,H 5 AT
-L,T,U,H 10 AT
-L,T,U,H 15 AT
SMC6 WT WT SR SR WT WT WT WT SR SR WT WT
SMC5 WT EQ WT EQ KI KI+EQ WT EQ WT EQ KI KI+EQ
Nse1 - - - - - - + + + + + +
Nse3 - - - - - - + + + + + +
Nse4 + + + + + + + + + + + +
Gal4AD SMC6
Gal4BD SMC5
Nse4
Gal4BD SMC5
Gal4AD SMC6
Nse3
Nse1
Nse4
D
1 2 3 4 5
Gal4BD SMC5
130kDa
100kDa
70kDa
55kDa
40kDa
35kDa
25kDa
Gal4BD
- WT EQ KI KI+EQ

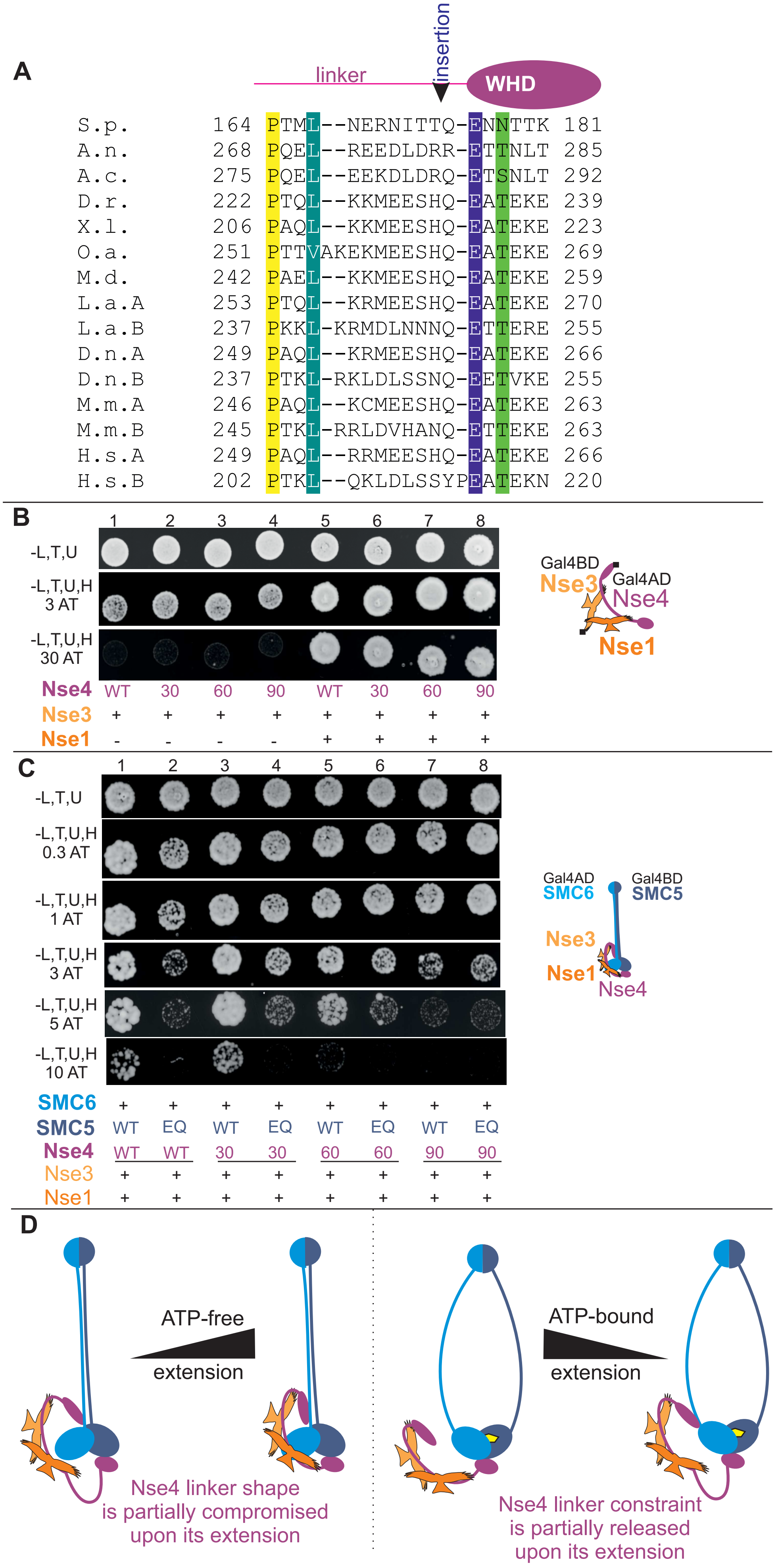
A
linker
insertion
WHD
S.p. 164 PTML--NERNITTQ-ENNTTK 181
A.n. 268 PQEL--REEDLDRR-ETTNLT 285
A.c. 275 PQEL--EEKDLDRQ-ETSNLT 292
D.r. 222 PTQL--KKMEESHQ-EATEKE 239
X.l. 206 PAQL--KKMEESHQ-EATEKE 223
O.a. 251 PTTVAKEKMEESHQ-EATEKE 269
M.d. 242 PAEL--KKMEESHQ-EATEKE 259
L.a.A 253 PTQL--KRMEESHQ-EATEKE 270
L.a.B 237 PKKL-KRMDLNNNQ-ETTERE 255
D.n.A 249 PAQL--KRMEESHQ-EATEKE 266
D.n.B 237 PTKL-RKLDLSSNQ-EETVKE 255
M.m.A 246 PAQL--KCMEESHQ-EATEKE 263
M.m.B 245 PTKL-RRLDVHANQ-ETTEKE 263
H.s.A 249 PAQL--RRMEESHQ-EATEKE 266
H.s.B 202 PTKL--QKLDLSSYPEATEKN 220
B
1 2 3 4 5 6 7 8
-L,T,U
-L,T,U,H 3 AT
-L,T,U,H 30 AT
Nse4 WT 30 60 90 WT 30 60 90
Nse3 + + + + + + + +
Nse1 - - - - + + + +
Gal4BD
Nse3
Gal4AD
Nse4
Nse1
C
1 2 3 4 5 6 7 8
-L,T,U
-L,T,U,H 0.3 AT
-L,T,U,H 1 AT
-L,T,U,H 3 AT
-L,T,U,H 5 AT
-L,T,U,H 10 AT
SMC6 + + + + + + + +
SMC5 WT EQ WT EQ WT EQ WT EQ
Nse4 WT WT 30 30 60 60 90 90
Nse3 + + + + + + + +
Nse1 + + + + + + + +
Gal4AD
SMC6
Gal4BD
SMC5
Nse3
Nse1
Nse4
D
ATP-free
extension
Nse4 linker shape is partially compromised upon its extension
ATP-bound
extension
Nse4 linker constraint is partially released upon its extension

Supplementary Table 1: SMC6(aa875-1024) peptide library

aa	Smc6 peptide sequence
875-899	TNILREKEAKKVQCAQVVADYTAKA
880-904	EKEAKKVQCAQVVADYTAKANTRCE
885-909	KVQCAQVVADYTAKANTRCERVPVQ
890-914	QVVADYTAKANTRCERVPVQLSPAE
895-919	YTAKANTRCERVPVQLSPAELDNEI
900-924	NTRCERVPVQLSPAELDNEIERLQM
905-929	RVPVQLSPAELDNEIERLQMQIAEW
910-934	LSPAELDNEIERLQMQIAEWRNRTG
915-939	LDNEIERLQMQIAEWRNRTGVSVEQ
920-944	ERLQMQIAEWRNRTGVSVEQAAEDY
925-949	QIAEWRNRTGVSVEQAAEDYLNAKE
930-954	RNRTGVSVEQAAEDYLNAKEKHDQA
935-959	VSVEQAAEDYLNAKEKHDQAKVLVA
940-964	AAEDYLNAKEKHDQAKVLVARLTQL
945-969	LNAKEKHDQAKVLVARLTQLLQALE
950-974	KHDQAKVLVARLTQLLQALEETLRR
955-979	KVLVARLTQLLQALEETLRRRNEMW
960-984	RLTQLLQALEETLRRRNEMWTKFRK
965-989	LQALEETLRRRNEMWTKFRKLITLR
970-994	ETLRRRNEMWTKFRKLITLRTKELF
975-999	RNEMWTKFRKLITLRTKELFELYLS
980-1004	TKFRKLITLRTKELFELYLSQRNFT
985-1009	LITLRTKELFELYLSQRNFTGKLVI
990-1014	TKELFELYLSQRNFTGKLVIKHQEE
995-1019	ELYLSQRNFTGKLVIKHQEEFLEPR
1000-1024	QRNFTGKLVIKHQEEFLEPRVYPAN

Supplementary Table 2: Primers used for PCR

SMC5 In-Fusion pGBKT7	oLV511	fw	CTGCATATGGCCATGGATGGCCTTAGGCCT
	oLV486	rev	GCCGCTGCAGGTCGACTTATGACGAAGAAATGAGTGC
Nse6 In-Fusion p416ADH1	EB77	fw	CCGCTCTAGAACTAGTATGAATGCGTCTAATAACATTTCAA
	EB78	rev	ATGACTCGAGGTCGACTTATCTTTTGTACGCTTGCC
Nse4 In-Fusion pGADT7	oLV575	fw	CAGATTACGCTCATATGTCCTCCATTGATAAACG
	oLV576	rev	CGAGCTCGATGGATCCTCAGCCATACCAAGTATTACTGT
insertion into p416ADH1	KB353	fw	CTTTAATTTGCGGCCGGGAGCTCGCCGGGATC
	KB354	rev	CTATAGGGCGAATTGG
$(G_4S)_6$ linker insertion	oLV579	fw	CATTACTACCGTCGAaGGTGGAGGAGGCTCT
	oLV580	rev	TATTTTCTTGGTCGACTGACCCTCCGCCT
3'end of Nse4	JP414	fw	CTCGAGATAACGCTTCGTAATTAAGAT
	JP415	rev	GTCGACCTTGCATAAATACTTAGTCC
Nse4 In-Fusion pGEM	oLV680	fw	TAGAACTAGTGGATCCATGTCCTCCATTGATAAACG
	oLV681	rev	GCTTGATATCGAATTCTCAGCCATACCAAGTATTACT
3xFLAG into *Nde*I	oLV691	fw	CTTGGTATGGCCATATGTCGTACGCTGCAGGT
	oLV692	rev	CTAGTGATTCACATACTACTTGTCATCGCCATCCT

Supplementary Table 3: Primers used for site-directed mutagenesis

SalI mutation in MCS	oLV522	fw	TCAAGCTTATCGATACCGTgGACCTCGAGTCATGTAATTAGT
	oLV523	rev	ACTAATTACATGACTCGAGGTCcACGGTATCGATAAGCTTGA
SalI insertion into Nse4	oLV520	fw	CTGAATGAACGTAACATTACTACCgtcgacCAAGAAAATAACACCACTAAAAATG
	oLV521	rev	CATTTTTAGTGGTGTTATTTTCTTGgtcgacGGTAGTAATGTTACGTTCATTCAG
NdeI insertion into Nse4	oLV689	fw	CTAACAGTAATACTTGGTATGGCcatatgTGAATCACTAGTGAATTCGAT
	oLV690	rev	ATCGAATTCACTAGTGATTCAcatatgGCCATACCAAGTATTACTGTTAG
Nse1 Q18A M21A	JP891	fw	GACAAGCATAAATTCATTCTTgcATATATAgcGTGTCGCACAGCTGGTGTTG
	JP892	rev	CAACACCAGCTGTGCGACACgcTATATATgcAAGAATGAATTTATGCTTGTC
Nse4 L62C	oLV291	fw	GAAGCAACCTTAGATGCTTTACTGtgTACTAAAACGGTTGATCTGGCTTC
	oLV292	rev	GAAGCCAGATCAACCGTTTTAGTAcaCAGTAAAGCATCTAAGGTTGCTTC
Nse4 T63R	oLV612	fw	AACCTTAGATGCTTTACTGCTTAgaAAAACGGTTGATCTGGCT
	oLV613	rev	AGCCAGATCAACCGTTTTtcTAAGCAGTAAAGCATCTAAGGTT
Nse4 K64C	oLV295	fw	GCAACCTTAGATGCTTTACTGCTTACTtgcACGGTTGATCTGGCTTCCA
	oLV296	rev	TGGAAGCCAGATCAACCGTgcaAGTAAGCAGTAAAGCATCTAAGGTTGC
Nse4 T65R	oLV614	fw	AGATGCTTTACTGCTTACTAAAAgGGTTGATCTGGCTTCCA
	oLV615	rev	TGGAAGCCAGATCAACCcTTTTAGTAAGCAGTAAAGCATCT
Nse4 V66C	oLV417	fw	GATGCTTTACTGCTTACTAAAACGtgTGATCTGGCTTCCATTAAAGC
	oLV418	rev	GCTTTAATGGAAGCCAGATCAcaCGTTTTAGTAAGCAGTAAAGCATC
Nse4 D67C	oLV297	fw	ATGCTTTACTGCTTACTAAAACGGTTtgTCTGGCTTCCATTAAAGCTAGG
	oLV298	rev	CCTAGCTTTAATGGAAGCCAGAcaAACCGTTTTAGTAAGCAGTAAAGCAT
Nse4 L68C	oLV377	fw	ACTGCTTACTAAAACGGTTGATtgcGCTTCCATTAAAGCTAGGCA
	oLV378	rev	TGCCTAGCTTTAATGGAAGCgcaATCAACCGTTTTAGTAAGCAGT
Nse4 L62C T65R	oLV676	fw	AGCAACCTTAGATGCTTTACTGtgTACTAAAAgGGTTGATCTGGCTTCCA
	oLV677	rev	TGGAAGCCAGATCAACCcTTTTAGTAcaCAGTAAAGCATCTAAGGTTGCT
Nse4 del87-91	oLV654	fw	TTGGAAGGCCCAAGTTTAATATTGAAATTAAGCAATTCCTCAACTATCC
	oLV655	rev	GGATAGTTGAGGAATTGCTTAATTTCAATATTAAACTTGGGCCTTCCAA
Smc5 E995Q	oLV494	fw	GCTCCGTTTCGAATAGTTGATcAAATAAATCAAGGAATGGATCCTC
	oLV495	rev	GAGGATCCATTCCTTGATTTATTTgATCAACTATTCGAAACGGAGC

Smc5 K57I	oLV674	fw	TTTGATTATCGGTCCAAATGGGACAGGTAtaAGCACAATTGTTTCAG
	oLV675	rev	CTGAAACAATTGTGCTtaTACCTGTCCCATTTGGACCGATAATCAAA
Smc6 L964A	oLV179	fw	GCTAGACTCACGCAAgcATTGCAAGCTTTAGAAGA
	oLV180	rev	TCTTCTAAAGCTTGCAATgcTTGCGTGAGTCTAGC
Smc6 L965A	oLV550	fw	TGCTAGACTCACGCAACTAgcGCAAGCTTTAGAAGAGACGT
	oLV551	rev	ACGTCTCTTCTAAAGCTTGCgcTAGTTGCGTGAGTCTAGCA
Smc6 Q966A	oLV552	fw	CTAGACTCACGCAACTATTGgcAGCTTTAGAAGAGACGTTAC
	oLV553	rev	GTAACGTCTCTTCTAAAGCTgcCAATAGTTGCGTGAGTCTAG
Smc6 A967C	oLV315	fw	AGACTCACGCAACTATTGCAAtgTTTAGAAGAGACGTTACGAAGGC
	oLV316	rev	GCCTTCGTAACGTCTCTTCTAAAcaTTGCAATAGTTGCGTGAGTCT
Smc6 L968A	oLV183	fw	GCAACTATTGCAAGCTgcAGAAGAGACGTTACGAAG
	oLV184	rev	CTTCGTAACGTCTCTTCTgcAGCTTGCAATAGTTGC
Smc6 E969A	oLV554	fw	ACGCAACTATTGCAAGCTTTAgcAGAGACGTTACGAAGGCGT
	oLV555	rev	ACGCCTTCGTAACGTCTCTgcTAAAGCTTGCAATAGTTGCGT
Smc6 E970A	oLV187	fw	CTATTGCAAGCTTTAGAAGcGACGTTACGAAGGCGT
	oLV188	rev	ACGCCTTCGTAACGTCgCTTCTAAAGCTTGCAATAG
Smc6 T971A	oLV556	fw	ACTATTGCAAGCTTTAGAAGAGgCGTTACGAAGGCGTAATG
	oLV557	rev	CATTACGCCTTCGTAACGcCTCTTCTAAAGCTTGCAATAGT
Smc6 L972A	oLV558	fw	TGCAAGCTTTAGAAGAGACGgcACGAAGGCGTAATGAAATGT
	oLV559	rev	ACATTTCATTACGCCTTCGTgcCGTCTCTTCTAAAGCTTGCA
Smc6 R973A	oLV189	fw	GCTTTAGAAGAGACGTTAgcAAGGCGTAATGAAATGTG
	oLV190	rev	CACATTTCATTACGCCTTgcTAACGTCTCTTCTAAAGC
Smc6 R974A	oLV191	fw	CTTTAGAAGAGACGTTACGAgcGCGTAATGAAATGTGGAC
	oLV192	rev	GTCCACATTTCATTACGCgcTCGTAACGTCTCTTCTAAAG
Smc6 R975A	oLV193	fw	GAAGAGACGTTACGAAGGgcTAATGAAATGTGGACCAAATTTC
	oLV194	rev	GAAATTTGGTCCACATTTCATTAgcCCTTCGTAACGTCTCTTC
Smc6 S1045R	oLV498	fw	AAGTCAGCGTTCAAGGATTAcgAGGGGGTGAAAAATCTTTTG
	oLV499	rev	CAAAAGATTTTTCACCCCCTcgTAATCCTTGAACGCTGACTT
